# C-reactive protein-induced injury in *Mycoplasma pneumoniae*-infected lung epithelial cells is mediated by the P38 MAPK/mitochondrial apoptosis pathway

**DOI:** 10.1128/spectrum.01626-24

**Published:** 2025-02-11

**Authors:** Lianjia Li, Yang Zhang, Lin Zhao, Yalin Shi

**Affiliations:** 1 Department of Pediatrics, Liaocheng Second People’s Hospital, Liaocheng, Shandong, China; 2 Department of Pediatric Surgery, Dongying People’s Hospital, Dongying, Shandong, China; 3 Department of Pediatrics, Weifang People’s Hospital, Weifang, Shandong, China; 4 Department of Pediatric Respiratory Medicine, Sunshine Unlon Hospital, Weifang, Shandong, China; Cinvestav-IPN, Mexico City, Mexico

**Keywords:** *Mycoplasma pneumoniae*, p38 MAPK/mitochondrial apoptosis pathway, apoptosis, C-reactive protein

## Abstract

Patients with *Mycoplasma pneumoniae* (MP) infections have markedly higher C-reactive protein (CRP). We investigated how CRP contributes to lung epithelial cell death following MP infection. CRP levels were assessed in children diagnosed with *Mycoplasma pneumoniae* pneumonia (MPP) and A549 lung epithelial cells infected with MP. A549 cells were genetically modified to overexpress CRP. Effects on cell viability, apoptosis, reactive oxygen species (ROS) and mitochondrial membrane potential (ΔΨm) were evaluated. The expression of proteins implicated in the p38 MAPK/mitochondrial apoptotic pathway was analyzed. The protective effects of the p38 MAPK inhibitor SB203580 and the mitochondrial protector cyclosporin A (CsA) were assessed. CRP levels were elevated in both MPP patients and MP-infected A549 cells compared to controls. Increased apoptosis and reduced cell viability were observed in MP-infected cells. CRP overexpression led to upregulation of proteins in the p38 MAPK/mitochondrial apoptosis pathway, increased cytoplasmic Cyt C, decreased Tom 20 and ΔΨm, and elevated ROS. Pretreatment with SB203580 or posttreatment with CsA reduced apoptosis and mitochondrial damage and enhanced cell survival. Increased CRP levels during MP infection promote lung epithelial cell death by activating the p38 MAPK/mitochondrial apoptosis pathway. Targeting this pathway could offer therapeutic potential to reduce lung damage in MPP patients.

**IMPORTANCE:**

This study provides critical information in understanding the pathophysiological mechanisms for MP infections concerning CRP in mediating lung epithelial cell injury. This study outlines the significant increase in MP-infected patients and shows its direct involvement in cell apoptosis through the p38 MAPK/mitochondrial apoptosis pathway. By explaining this pathway, the possibility of targeting CRP and its connected signaling mechanisms to devise therapeutic interventions for the amelioration of lung damage in MP-infected patients is brought to light. The implications of such data are not merely in the added knowledge for disease pathobiology but also it brings new promise for novel intervention strategies to result in improved clinical outcomes. The elucidation of specific molecular targets inside the apoptosis pathway heralds a new area regarding the direction of future research and clinical application for humanity in general and concerning the broader relevance and impact of this study on respiratory diseases.

## INTRODUCTION

*Mycoplasma pneumoniae* (MP) is a major pathogen that causes respiratory tract infections in humans (1). Over the past decade, nationwide surveillance of acute respiratory infections has identified MP as the leading atypical pathogen among Chinese children aged 5 to 7 (2). *Mycoplasma pneumoniae* pneumonia (MPP) caused by MP infection is a frequent respiratory ailment in children. *M. pneumoniae* causes community-acquired pneumonia (CAP) (3, 4), and its infection begins with bacterial adherence to the surface of airway epithelial cells, followed by bacterial multiplication and colonization (5). The clinical symptoms of MP-related respiratory illnesses are attributable to the bacterium’s cytoadhesion to respiratory cells, which contributes to persistent infection and protection from mucociliary clearance (6). MP cytadherence activates the inflammasome, leading to the production of pro-inflammatory cytokines from infected cells, thereby contributing to disease progression (7, 8).

C-reactive protein (CRP) indicates systemic inflammatory response and has been linked to the pathological progression of atherosclerosis and other inflammatory diseases (9, 10). Clinical investigations have demonstrated that elevated CRP levels in MP patients predict the severity of pneumonia (11, 12). Elevated CRP levels, along with serum amyloid A and procalcitonin, have been observed in children with MP infection, demonstrating their role in disease progression (13).

Apoptosis is a controlled cell death process and an important homeostatic mechanism (14). Mitochondria are essential for maintaining cellular energy balance, regulating apoptosis and modulating redox signaling. Mitochondrial damage can cause the production of pro-apoptotic proteins, resulting in defective apoptosis and contributing to a variety of illnesses (15). The activation of these proteins leads to permeabilization of the mitochondrial membrane, facilitating the release of cytochrome c and versatile apoptotic factors. Cytochrome c subsequently binds with apoptotic protease activating factor-1 (Apaf-1) and pro-caspase-9, which in turn activates caspase-3, ultimately resulting in apoptosis (16–18). It has been reported that CRP can induce human coronary vascular smooth muscle cells apoptosis via ROS production by NOX4 (19). CRP can increase ROS production in cardiomyocytes and induce mitochondrial damage (20). Moreover, previous research has shown that the p38 MAPK pathway further enhances mitochondrial apoptosis (21, 22). The MAPK family plays roles in transcription factor activation, cell proliferation and even necrosis (23, 24). p38 MAPK has been revealed to regulate gene expression involved in cell cycle arrest and apoptosis (25). By activating Bax, p38 MAPK induces cell death (26, 27), thus underscoring its pivotal role in apoptosis regulation.

The pathogenic processes of MP are intricate, and specific mechanisms underlying these pathological events remain unclear. The primary objective of this research is to explore how CRP affects *M. pneumoniae*-infected lung epithelial cells to better understand the infection’s pathophysiology and identify potential therapeutic targets. Given the established link between elevated CRP levels and the severity of MP infection, we investigated the mechanistic role of CRP in modulating inflammatory responses and apoptotic pathways in lung epithelial cells infected with MP. This question is significant as it addresses the gap in understanding how systemic inflammatory markers like CRP contribute to local cellular responses during MP infection. Understanding this mechanism could provide insights into the progression of MP-related respiratory illnesses and potential therapeutic interventions against this infection.

## MATERIAL AND METHODS

### Collection of samples

Our hospital enrolled 20 children diagnosed with MPP-related respiratory infections between January 2018 and January 2019. The patients were in the age range of 2–7 years, with a total of nine males and 11 females. Diagnosis of MPP was based on the presence of specific symptoms: (1) positive serum *Mycoplasma pneumoniae*-specific IgM antibody with titer ≥ 1:160; (2) respiratory samples with a *Mycoplasma pneumoniae* DNA copy number of at least 500; and (3) absence of other known causes of pneumonia. Following serological diagnosis of MP infection, polymerase chain reaction (PCR) was used to confirm viral presence in nasal swabs. Twenty healthy children (age range, 2–7 years) served as a comparison group selected based on similarity to study participants but without immune system abnormalities or chronic infectious conditions. 2 mL of blood samples was obtained from both groups for CRP expression analysis and concentration determination via immunoturbidimetric CRP testing. Additionally, protein extraction from blood samples was performed using standard lysis protocols, and western blot analysis was conducted to evaluate CRP protein expression. Approval for this research was obtained from the Institutional Human Ethical Committee of Sunshine Unlon Hospital (No.: SN2018-015), and parental or legal permission forms were completed for all participating children.

### Immunoturbidimetric assay

CRP was detected by an immunoturbidimetric assay (Roche Diagnostics, Ltd., UK). Briefly, CRP concentrations in venous blood samples collected from both the MPP-infected children and the healthy comparison group were determined. This method relies on the principle of measuring the increase in turbidity caused by the formation of antigen–antibody complexes. For the serum test, 50 µL of the primary sample was mixed with 200 µL of Atellica CH Diluent in a dilution tube. Afterward, 40 µL of reagent 1 (containing glycine, sodium chloride, EDTA, and sodium azide), 4 µL of the diluted sample, and 40 µL of reagent 2 (CRP antibody with sodium azide) were sequentially added. The mixture was incubated at 37°C, and absorbance was measured at 571 nm. The serum CRP concentration was determined using a calibration curve generated from Atellica CH CRP_2 CAL, assessing the inflammatory response associated with MPP infection.

### Cell culture, MP culture, and cell infection

A549 human lung adenocarcinoma cells were grown in RPMI 1640 media. *Mycoplasma pneumoniae* was cultivated at 37°C in SP4 media, and its concentration in the broth medium was assessed using colony-forming units and color-changing units (CCUs). To infect the A549 cells, *Mycoplasma pneumoniae* was added to the culture at a multiplicity of infection of 10, which involved mixing 1 mL of MP with 10 mL of RPMI 1640 media, resulting in approximately 1 × 10^7^ CFU/10^6^ cells and incubated for 12 h.

### Cell transfection

Cell transfection was conducted using A549 cells (5 × 10^5^/well) seeded in a 12-well plate. Lipofectamine 2000 (Invitrogen, USA) was employed as the transfection reagent. Upon reaching a fusion rate of 95–100%, recombinant plasmid pTracer CMV2-CRP or pTracer CMV2 containing CRP or control, respectively, was utilized for transfection. The transfection mixture consisting of the plasmid and Lipofectamine 2000 was added to the cells. After transfection, the cells were transferred to an incubator for further cultivation under appropriate conditions for 24 h. This protocol adhered to the manufacturer’s guidelines for Lipofectamine 2000-mediated transfection and was tailored to the specific requirements of A549 cell culture, facilitating the investigation of CRP function and expression levels within the cellular context.

### Western blot

Protein extraction was performed using cell lysis buffer. The cells were lysed in ice-cold RIPA buffer with protease inhibitors (Thermo Fisher, USA), then centrifuged at 12,000 rpm for 12 min. Protein extraction was carried out from both blood plasma and cell lysates using RIPA buffer containing protease inhibitors (Thermo Fisher, USA). Membranes were blocked with 5% non-fat dry milk in Tris-buffered saline with 0.1% Tween-20 for 1 h at room temperature. The primary antibodies included: CRP (Abcam, ab68743, 1:2000), Caspase 3 (CST, 9662, 1:1200), Cleaved Caspase 3 (Millipore #AB3623, 1:800), PARP (Cell Signaling Technology, 9542, 1:500), Cleaved PARP (Santa Cruz, 56196, 1:200), Bcl-2 (Proteintech #68103–1, 1:500), Bax (Proteintech, 50599–2, 1:400), Rac1 (CST, 2465, 1:1000), Phospho-Rac1 (Merck Millipore, 07–896, 1:100), ACTIB (Abcam, ab8227), TAK1 (Abcam, ab109526, 1:1000), Phospho-TAK1 (Cell Signaling Technology, 4536, 1:400), MKK3 (Santa Cruz Biotechnology, sc-271283, 1:50), Phospho-MKK3 (Sigma-Aldrich, SAB4504588, 1:100), P38MAPK (Thermo Fisher Scientific, 44–684G, 1:200), Cytochrome C (Sigma-Aldrich, C7732, 1:250), APAF-1 (Santa Cruz Biotechnology, sc-13541, 1:20), Caspase 9 (Thermo Fisher Scientific, PA1-29157), Cleaved Caspase 9 (Santa Cruz Biotechnology, sc-56076, 1:200), and Beta-actin (Thermo Fisher Scientific, MA5-15739). Next day, the membranes were incubated with secondary antibodies for 1 h, including anti-mouse (Thermo Fisher Scientific, 31430, 1:2000) and anti-rabbit (Santa Cruz Biotechnology, sc-2357, 1:500). The membranes were again rinsed with PBS, and protein expression was detected using an ECL Detection Kit (Beyotime, China).

### QRT-PCR analysis

RNA was extracted using TRIzol reagent (Invitrogen, USA) and reverse-transcribed into cDNA using the Takara RNA PCR Kit (Takara, Japan). Quantitative RT-PCR was performed using FastStart Universal SYBR-Green Master (Roche, CH) on an appropriate thermal cycler. The 2^ΔΔCt^ method was employed to compare the levels of gene expression between samples, ensuring accurate quantification. For the quantitative RT-PCR, the reaction mixture included SYBR-Green Master, cDNA template, specific primers and nuclease-free water. The relative expression levels of CRP were calculated by normalizing the Ct values to a housekeeping gene and comparing them to a control sample. Primer sequences for CRP were Fwd primer: 5′- TCGACCCGTGGGTACAGTATT-3′ and Rev primer: 5′- TTTGGACCGTTTCCCAGCAT-3′.

### Nuclear staining

Cells (2 × 10^4^) were cultured in a 96-well plate until they covered 70–80% of the well’s bottom. For nuclear staining, 30 µL of DAPI was supplemented to each well for 5 min. Fluorescence microscope (Olympus IX51, Japan) was used to examine the nuclear morphology, where apoptotic cells exhibited a dense nuclear staining or staining of dense fragments. The results were analyzed using ImageJ software (version 1.53, National Institutes of Health, USA).

### Immunofluorescent assay

Cells were grown to 70–80% confluency and then incubated with primary antibodies at 4°C. The primary antibodies used included CRP (1:1000, Abcam, catalog #ab68743), Tom 20 (1:500, Santa Cruz Biotechnology, catalog #sc-11415) and Cyt C (1:500, Sigma-Aldrich, catalog #C7732). Following the primary antibody incubation, secondary antibodies conjugated to fluorophores (Invitrogen, catalog #A-11001 or #A-11012) were applied to the cells for at least half an hour. The nuclei were then stained with DAPI (Thermo Fisher Scientific, catalog #D1306) by adding 30 µL to each well and incubating for 5 min. The signals in the cells were observed using a laser scanning microscope (FV3000, Olympus Corporation, Japan). The results were analyzed using ImageJ software (version 1.53, National Institutes of Health, USA).

### MTT assay

A549 cells were cultured in plates until they reached 70–80% confluence. After reaching the desired cell density, the supernatant was carefully aspirated from each well to remove any excess medium. At the completion of each incubation time point (12, 24, 48, and 72 h), 10 µL of 5 mg/mL MTT solution was added to each well, followed by an additional 4 h of incubation. After that, 150 µL of DMSO was added to the cells in order to dissolve the formazan crystals. Using a microplate reader (Multiskan MK3, Thermo Fisher Scientific, USA), the absorbance at 490 nm was measured to assess cell viability.

### Flow cytometry

Cells (1 × 10^6^) were seeded in six-well plates while they were still undergoing rapid division. The cells were extracted and resuspended in a binding buffer that contained propidium iodide (PI) and Annexin V-FITC. The samples were then analyzed using a BD FACSCelesta flow cytometer (BD Biosciences, USA). Data acquisition was followed by analysis using FlowJo software (FlowJo 10.8.1, BD Biosciences, USA).

### ROS detection

Reactive oxygen species (ROS) levels in A549 cells were assessed using the fluorescent probe DCFH-DA. A549 cells (5 × 10^3^) were seeded into 48-well plates and treated with 10 µmol/L DCFH-DA and incubated for 20 min. After incubation, the fluorescence intensity was measured using a fluorescence microscope (Olympus IX51, Japan) to examine the content of ROS.

### Mitochondrial membrane potential (ΔΨm)

The JC-1 Kit (Beyotime, China) was used to evaluate the mitochondrial membrane potential (ΔΨm). A549 cells were cultured into culture vessels and incubated until they reached the desired confluency. The cells were subsequently treated with JC-1 (20 µg/mL) for a duration of 30 min, followed by a thorough washing to eliminate any surplus dye. The alterations in ΔΨm were observed using confocal laser scanning microscopy (FV3000, Olympus Corporation, Japan). The JC-1 dye has the ability to accumulate in mitochondria, and its accumulation is reliant on the potential, as seen by a change in fluorescence emission from green (JC-1 monomers) to red (JC-1 aggregates). The excitation/emission wavelengths for detecting JC-1 monomers are 490/530 nm, and for detecting JC-1 aggregates, the wavelengths are 525/590 nm. The red/green fluorescence intensity ratio serves as an indication of ΔΨm and decrease in the ratio indicates a loss of ΔΨm.

### Statistics

All data were presented as means and standard deviations. Statistical analyses were conducted using GraphPad Prism (version 8.0, USA). Statistical significance between groups was tested using one-way analysis of variance, and each experiment was carried out at least three times.

## RESULTS

### Enhanced CRP expression in children with MPP and MP induced up-regulation of CRP in A549 cells

**Fig 1:**
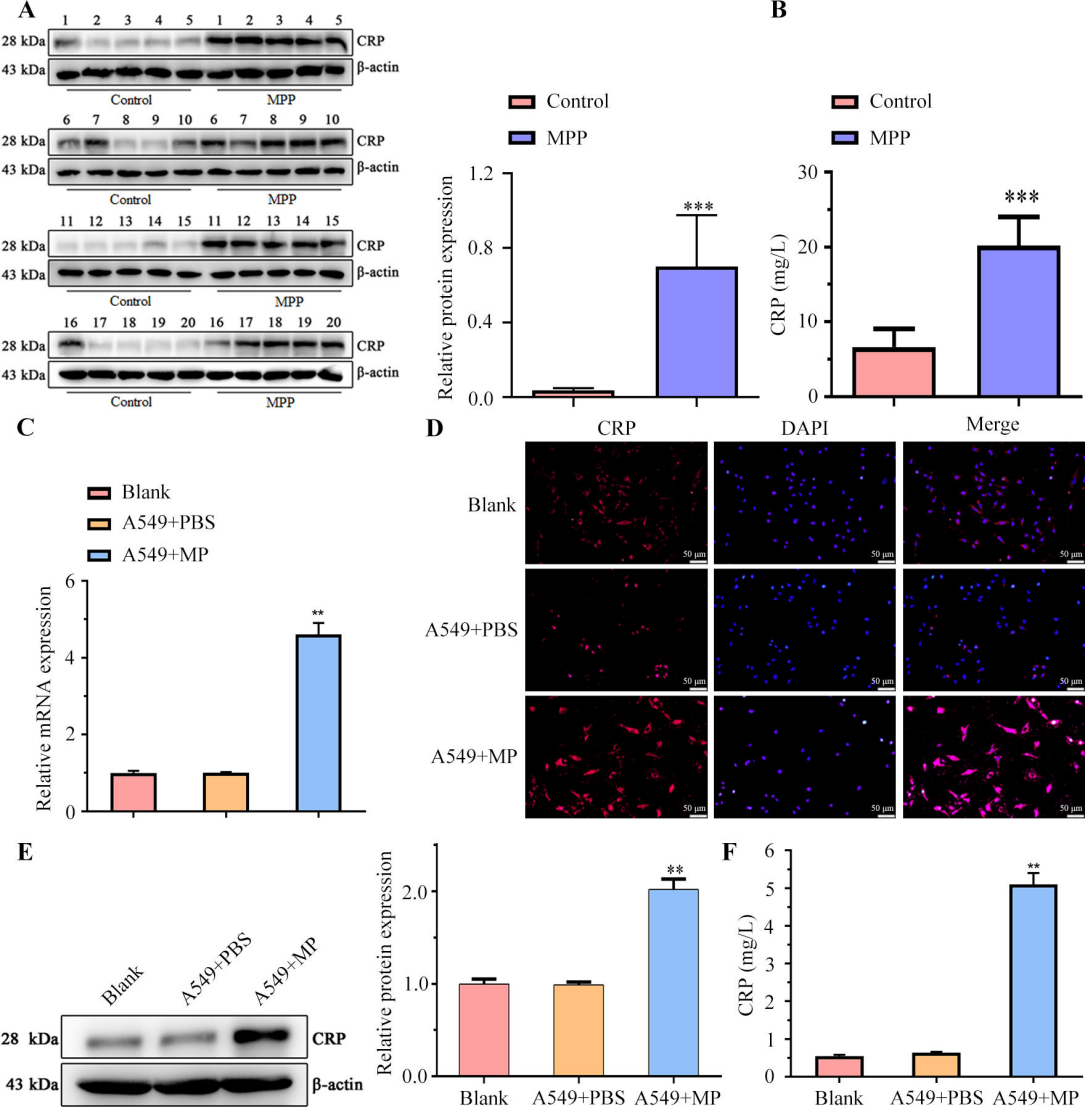
Upregulated CRP Levels in the blood samples of children with MPP and MP. (**A**) CRP expression levels in the blood samples of 20 children from the MPP and control groups. (B) Immunoturbidimetric measurement of CRP levels in the blood samples of 20 children from MPP and control groups separately. (**C**) CRP expression in A549 cells under variable treatments (Blank, A549 + PBS, A549 + MP) (*n* = 3/group) was detected by qRT-PCR, (**D**) immunofluorescent assay, and (E) Western blot. (**F**) Immunoturbidimetric assay to detect CRP expression in Blank, A549 + PBS and A549 + MP cells (*n* = 3/group). All experiments were repeated three times. **P < 0.01; ***P < 0.001.

We initially investigated CRP levels in the blood samples of children and compared them between those with MPP (the MPP group) and healthy controls (the control group) using immunoturbidimetry and western blot assay. Remarkably, we observed elevated CRP concentrations in all 20 cases within only the MPP group (Fig. 1A). Next, the CRP expression level in the blood of 20 children in each group was measured by immunoturbidimetry, and we found upregulated CRP level in MPP children (Fig. 1B). Subsequently, we transfected respiratory epithelial cells, A549 cells, with MP to assess whether MP could induce upregulation of CRP levels in these cells. QRT-PCR indicated enhanced levels of MP in transfected cells as compared to control and PBS group demonstrating successful transfection of MP in these cells (Fig. 1C). The results of IF on these cells revealed increased expression of CRP in the A549 + MP group (Fig. 1D). Furthermore, heightened CRP levels were observed in cells transfected with MP (Fig. 1E). Lastly, immunoturbidimetric assay demonstrated that A549 cells expressed elevated levels of CRP in the presence of MP (Fig. 1F). Overall, these results suggest a significant association between MPP infection and increased CRP expression.

### MP infection effected cells viability, apoptosis and ROS production

**Fig 2:**
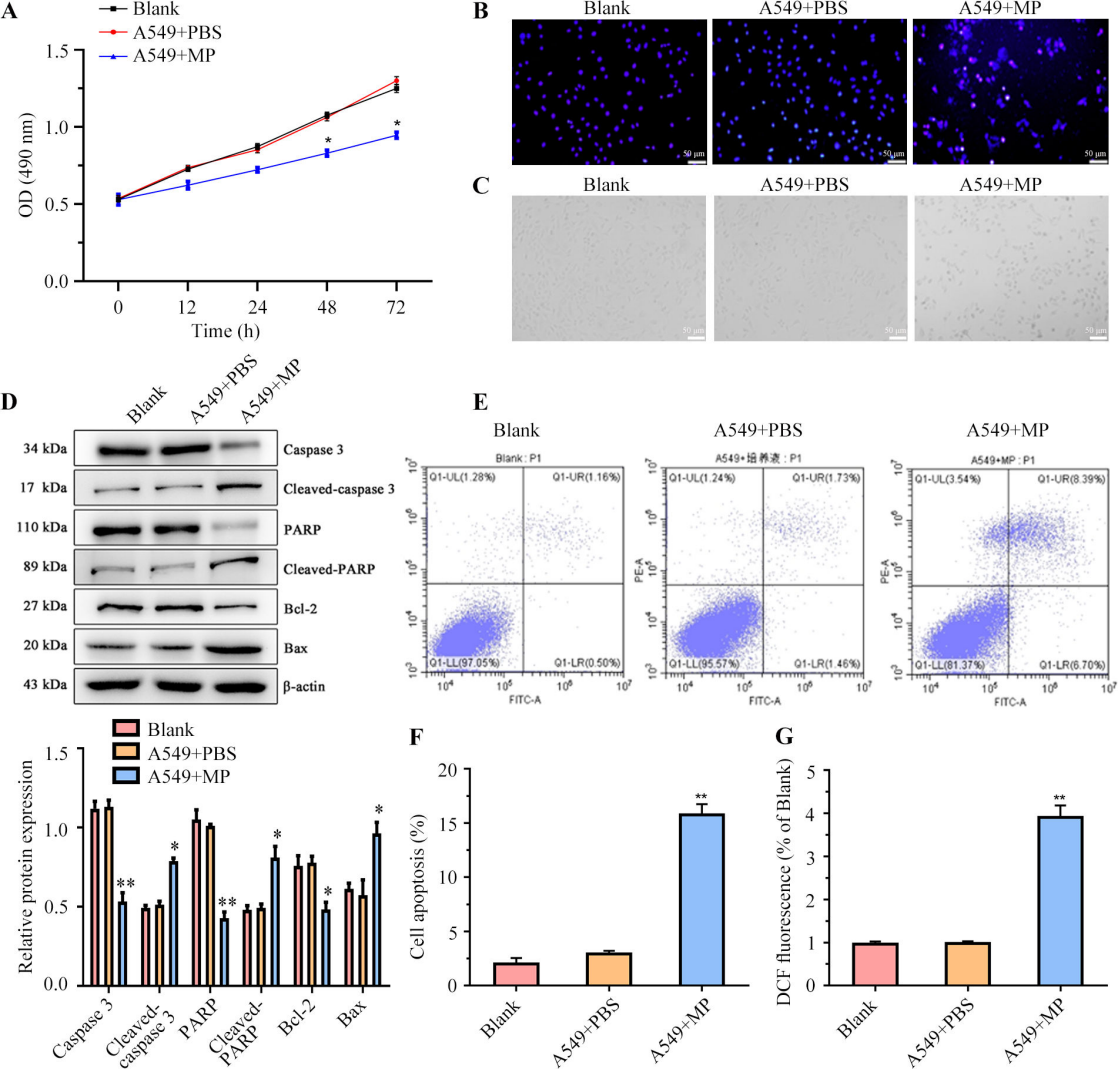
Impact of MP infection on A549 cell viability, apoptosis and ROS production.(**A**) Cell viability of A549 cells was accessed. (**B**) IF imaging was employed to observe nuclear morphology in A549 cells. (**C**) Inverted microscope was used to detect the morphological changes in A549 cells. (**D–F**) Western blot analysis and flow cytometry were conducted to detect the expressions of apoptotic proteins in A549 cells. (**G**) DCFH-DA method was employed to measure the ROS content in A549 cells. Three samples per group. All experiments were repeated three times. *P < 0.05; **P < 0.01.

We proceeded to assess the impact of CRP on A549 cells by evaluating their viability, apoptosis and ROS production. MTT test results indicated a substantial reduction in cell viability in the A549 + MP group compared to the Blank and A549 + PBS groups (Fig. 2A). Additionally, nuclear morphology analysis revealed alterations in nuclei of A549 + MP cells characterized by smaller size and more compact chromatin, resulting in an overall volume reduction of cells (Fig. 2B and C). Furthermore, we investigated the effect of MP transfection on apoptosis. Western blot analysis revealed markedly elevated expression levels of cleaved caspase-3, cleaved PARP, and Bax, accompanied by suppression of Bcl-2 expression in the A549 + MP group (Fig. 2D). Flow cytometry analysis corroborated these findings, showing a notably higher apoptotic rate in the A549 + MP group (Fig. 2E and F). Finally, DCFH-DA data demonstrated increased ROS levels in the A549 + MP group compared to cells without MP transfection (Fig. 2G). Overall, these findings indicate that MP infection reduces cell viability and promotes apoptosis in A549 cells.

### CRP activated p38 MAPK/mitochondrial pathway

**Fig 3:**
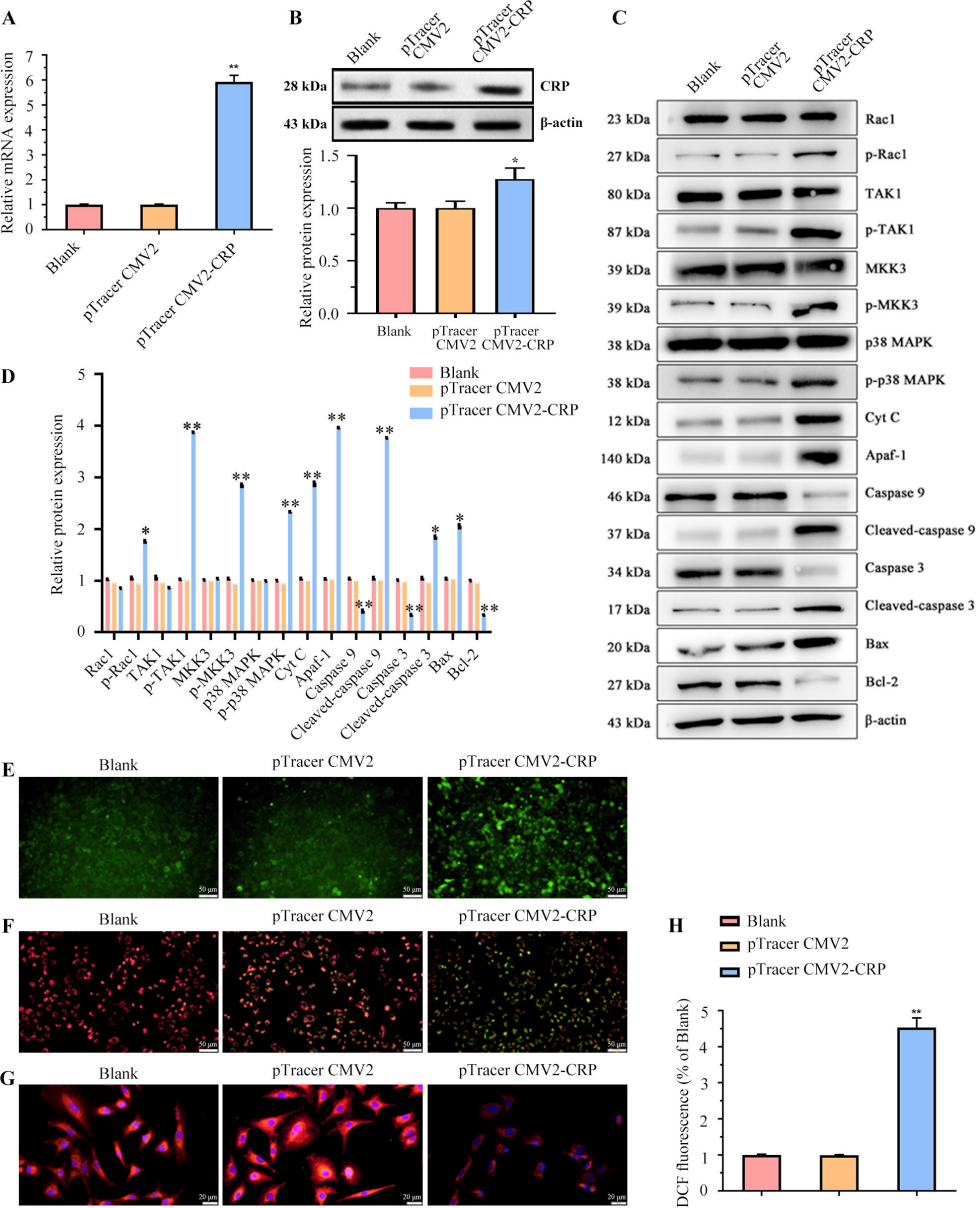
CRP activated p38 MAPK/Mitochondrial apoptosis pathway. (**A**) CRP expression in A549 cells in the Blank, pTracer CMV2, and pTracer CMV2-CRP groups was analyzed by qRT-PCR and (B) western blot. (**C**) Western blot detection of p38 MAPK and mitochondrial apoptosis-related protein expressions in A549 cells and the statistical graph (D). (**E**) Immunofluorescence observation of Cyt C distribution in A549 cells. (**F**) IF indicates changes in ΔΨm in A549 cells. (**G**) IF detects Tom 20 expression levels in A549 cells. (**H**) DCFH-DA assay to detect ROS content in A549 cells. Three samples per group. All experiments were repeated three times. *P < 0.05; **P < 0.01.

To examine the mechanistic role of CRP, we constructed a recombinant plasmid to overexpress CRP in A549 cells and grouped them into the Blank, pTracer CMV2, and pTracer CMV2-CRP groups. Transfection efficiency was verified by qRT-PCR and western blot, showing significant CRP overexpression in the pTracer CMV2-CRP group compared to the Blank and pTracer CMV2 groups (Fig. 3A and B). Next, we examined the protein expression of MAPK-dependent pathway proteins, as previous investigations have demonstrated that monomeric CRP induces damage and death of human coronary artery endothelial cells via a p38 MAPK-dependent mechanism (28, 29). Our results revealed significantly increased expression levels of p38 MAPK pathway-related proteins (p-Rac1, p-TAK1, p-MKK3 and p-p38 MAPK) and all mitochondrial apoptosis-related proteins (Apaf-1, Cyt C, c-caspase 3, c-caspase 9 and Bax), while Bcl-2 expression was decreased (Fig. 3C and D). We then explored whether CRP could cause mitochondrial damage and trigger the mitochondrial death pathway in A549 cells. In the pTracer CMV2-CRP group, we observed a substantial secretion of Cyt C from mitochondria into the cytoplasm (Fig. 3E). JC-1 staining revealed that the ΔΨm was lowest in the pTracer CMV2-CRP group compared to the Blank and pTracer CMV2 groups (Fig. 3F). Additionally, the expression level of Tom 20, an important parameter of mitochondrial function, was clearly reduced in the pTracer CMV2-CRP group (Fig. 3G). Moreover, ROS content detection showed that CRP overexpression significantly increased ROS levels in A549 cells (Fig. 3H). These findings imply that CRP may cause cell death via the mitochondrial apoptosis mechanism.

### CRP reduced cell viability and triggers apoptosis via the p38 MAPK/mitochondrial apoptosis pathway

**Fig 4:**
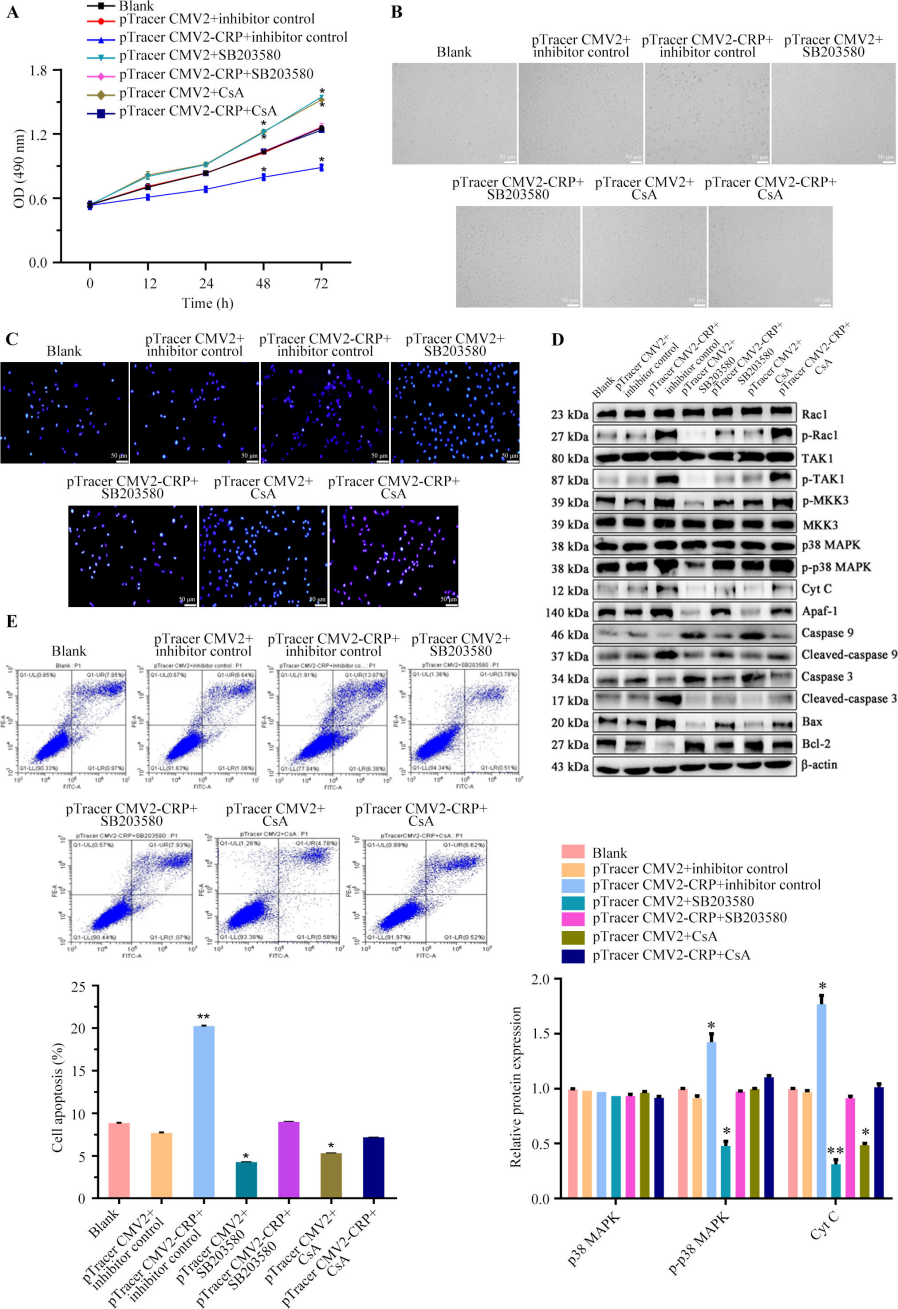
CRP reduced cell viability and triggers apoptosis by activating p38 MAPK/mitochondrial apoptosis pathway. (**A**) Cell viability of A549 cells was detected by MTT assay. (**B, C**) Morphology of A549 cells and its nucleus was observed under an inverted microscope. (**D**) Protein expressions of p38 MAPK and mitochondrial apoptosis-related pathway proteins in A549 cells were detected. (**E**) Apoptosis of A549 cells was detected by flow cytometry. *n* = 3. All experiments Fig. 4 were repeated three times. *P < 0.05; **P < 0.01.

**Fig 5:**
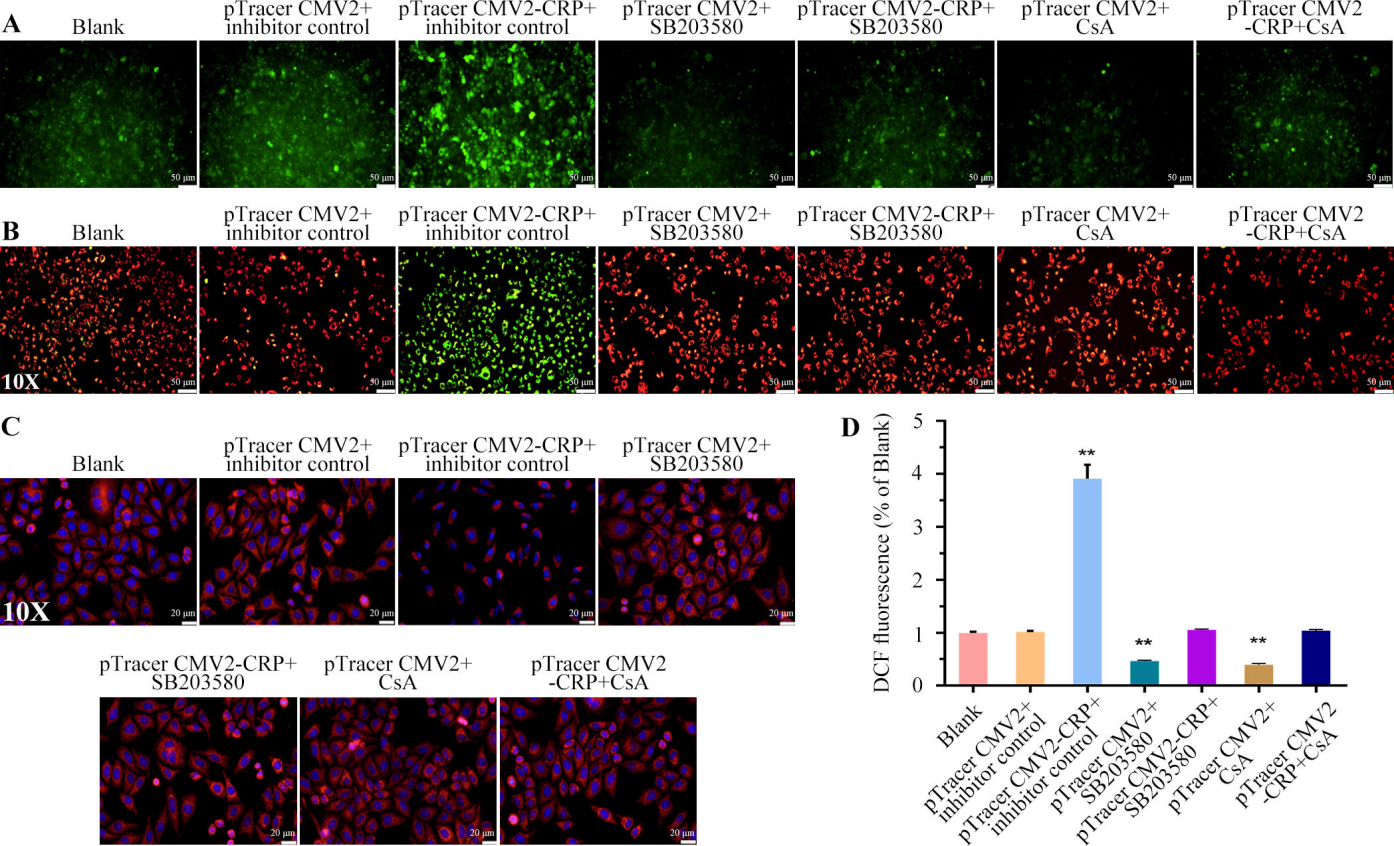
CRP induced mitochondrial damage by activating the p38 MAPK/Mitochondrial apoptosis pathway. (A) Distribution of Cyt C in A549 cells was observed using IF. (B) Changes in ΔΨm in A549 cells after different treatments were observed using IF. (C) Expression level of Tom 20 in A549 cells was observed using IF. (D) ROS content in A549 cells was detected by DCFH-DA method. Three samples per group, and all experiments were repeated three times. **, *P*<0.01.

To investigate whether CRP causes apoptosis in pulmonary epithelial cells via the p38 MAPK/mitochondrial pathway, we performed rescue experiments using the p38 MAPK inhibitor SB203580 and the mitochondrial protector CsA. A549 cells were divided into the following groups: Blank, pTracer CMV2 + inhibitor control, pTracer CMV2-CRP + inhibitor control, pTracer CMV2 + SB203580, pTracer CMV2-CRP + SB203580, pTracer CMV2 + CsA, and pTracer CMV2-CRP + CsA. MTT experiment demonstrated a notable reduction in cell viability in the pTracer CMV2-CRP + inhibitor control group. However, cell viability increased significantly with the addition of SB203580 or CsA (Fig. 4A). This suggests that SB203580 or CsA attenuates the CRP-induced reduction in cell viability. Observation of cell and nuclear morphology showed reduced cytoplasmic volume and significant shrinkage of cells and nuclei in the pTracer CMV2-CRP + inhibitor control group. In contrast, cells in the pTracer CMV2 + SB203580 and pTracer CMV2 + CsA groups exhibited improved cellular growth, indicating that SB203580 or CsA mitigated the effects of CRP on cell morphology (Fig. 4B and C). Moreover, increased expression levels of p-Rac1, p-TAK1, p-MKK3, p-p38 MAPK, Cyt C, Apaf-1, cleaved-caspase 9, cleaved-caspase 3, and Bax, and decreased levels of Bcl-2 in the pTracer CMV2-CRP + inhibitor control group were observed (Fig. 4D). The addition of SB203580 or CsA moderated these CRP-induced changes in protein expression, suggesting that they mitigate CRP’s influence on apoptosis-related proteins. Further, flow cytometry showed a significantly higher apoptotic rate in the pTracer CMV2-CRP + inhibitor control group. This rate was significantly reduced in the pTracer CMV2 + SB203580 and pTracer CMV2 + CsA groups, indicating that SB203580 or CsA weakened CRP-induced apoptosis (Fig. 4E). We assessed mitochondrial damage by measuring cytoplasmic Cyt C content, changes in ΔΨm, expression of Tom 20, and ROS content. The pTracer CMV2-CRP + inhibitor control group showed increased cytoplasmic Cyt C and ROS content and decreased ΔΨm and Tom 20 levels (Fig. 5). Inhibition of the p38 MAPK pathway significantly reversed these mitochondrial abnormalities, similar to the effects observed with CsA treatment. Overall, these findings show that CRP overexpression activates the p38 MAPK/mitochondrial apoptosis mechanism, leading to suppressed cell viability, increased apoptosis, and mitochondrial dysfunction in A549 cells. The addition of SB203580 or CsA effectively mitigates these effects, highlighting their potential protective roles.

## DISCUSSION

MP is a major causative agent of respiratory tract infections, particularly MPP, which has a high incidence in children (30). Adhesion is crucial for MP pathogenicity, as it attaches to the bronchial ciliary epithelium, causing metabolic and ultrastructural changes in infected respiratory cells (31). Despite these insights, the precise mechanisms underlying MP infection remain unclear (32), highlighting the need for reliable biomarkers and an understanding of the involved processes.

CRP is produced during inflammation in the human body (33, 34), and high levels of CRP are associated with bacterial infections, even in children (35). This sharp increase in CRP is a precursor to a full-blown MP respiratory infection in children (36, 37). We showed the enhanced expression level of CRP in the blood of children with MP infection. Similarly, *in vitro* experiments showed that CRP expression was considerably elevated in MP-infected A549 cells. Previous literature has indicated that CRP is responsible for inflammation in human vascular smooth muscle cells (19), and CRP/ROS-mediated damage to pulmonary epithelial cells was enhanced by Corexit 9500A (38, 39). CRP has also been shown to trigger apoptosis in various tissues, including umbilical vein endothelial cells, monocytes, and HL-1 heart muscle cells (40–42). In our study, we found that both MP-infected and CRP-overexpressed A549 cells had dramatically elevated apoptotic rates. Our findings suggest that MP infection negatively impacts the morphology of A549 cells. High CRP expression may indicate MP infection, and CRP-induced apoptosis could play a role in the damage MP inflicts on lung epithelial cells.

The p38 MAPK pathway is a crucial component of the MAPK family primarily involved in regulating inflammatory responses, differentiation, and apoptosis (43–45). The p38 MAPK pathway activates p53, which in turn induces the pro-apoptotic protein Bax to produce cytochrome c, leading to the activation of caspase-3 (46, 47). Moreover, ROS regulated the expression of p38 MAPK, resulting in mitochondrial damage and forming a feedback loop that exacerbates the damage (48). The p38 MAPK pathway is implicated in mitochondrial pathway-related cell apoptosis of oviduct magnum epithelial cells (49), and monomeric CRP promoted injury and apoptosis of human coronary artery endothelial cells through a p38 MAPK-dependent pathway. Our findings align with these studies, showing that elevated CRP levels activated the p38 MAPK pathway to induce apoptosis in A549 cells. Furthermore, we found that the p38 MAPK inhibitor SB203580 was able to restore A549 cells exposed to CRP, preventing apoptosis. These results indicate that CRP influences pulmonary epithelial cells through the p38 MAPK pathway.

Mitochondria, as the center of energy metabolism, are closely linked to cell apoptosis, with the depletion of ΔΨm being an early event in the apoptotic cascade (50, 51). Stimulation has been shown to induce mitochondrial dysfunction, leading to membrane permeabilization or rupture, as indicated by the depolarization of Δψm. This causes the release of CytC into the cytoplasm, where it binds with Apaf-1, recruits caspase 9, and activates caspase 3, resulting in apoptosis (52). Additionally, Cao et al. reported that the p38MAPK signaling pathway mediates oleuropein-induced apoptosis via the mitochondrial apoptotic cascade in A549 cells (53). CRP can increase ROS production by NOX, leading to apoptosis of vascular smooth muscle cells (54). Furthermore, CRP promotes the phosphorylation of p38 MAPK, contributing to elevated ROS levels in multiple myeloma, which further exacerbates cellular stress and apoptosis (55). Our data showed that following MP infection or CRP overexpression, c-caspase 3 and Bax were strongly elevated, whereas Bcl-2 was strongly downregulated, indicating that CRP up-regulation induced mitochondrial apoptosis. Activation of p38 MAPK also leads to mitochondrial damage, including an increase in Cyt C production, a reduction in ΔΨm, and apoptosis, along with decreased Tom 20 expression. These findings are consistent with western blot studies showing mitochondrial apoptotic events in A549 cells with CRP overexpression, including increased Bax expression, suppression of Bcl-2, Bax insertion into the mitochondrial outer membrane, Cyt C release, and cleavage of caspases 3 and 9. Furthermore, our study demonstrated that the use of MAPK pathway inhibitors or mitochondrial protectors partially reversed the apoptosis and mitochondrial damage induced by CRP, suggesting that CRP promotes apoptosis in A549 cells via the p38 MAPK/mitochondrial pathway.

However, this study had limitations, including a small sample size and the lack of differentiation between CRP subtypes. Future studies should expand the sample size, explore the distinct roles of CRP isoforms, and investigate additional pathways by which CRP regulates apoptosis, particularly in respiratory infections associated with MP.

### Conclusion

Our study found that MP upregulated CRP production and CRP caused apoptosis of A549 cells through the p38 MAPK/mitochondrial apoptosis pathway. These findings highlight the impact of CRP on lung epithelial cells and may provide new insights into the mechanisms by which MP induces lung infection, suggesting potential therapeutic targets for treating MP-related respiratory infections.
